# Exploring the Association Between Multidimensional Dietary Patterns and Non-Scarring Hair Loss Using Mendelian Randomization

**DOI:** 10.3390/nu17152569

**Published:** 2025-08-07

**Authors:** Lingfeng Pan, Philipp Moog, Caihong Li, Leonard Steinbacher, Samuel Knoedler, Haydar Kükrek, Ulf Dornseifer, Hans-Günther Machens, Jun Jiang

**Affiliations:** 1 Clinic for Plastic, Reconstructive and Hand Surgery, Klinikum Rechts der Isar, Technical University of Munich, 81675 Munich, Germany; lingfeng.pan@tum.de (L.P.); philipp.moog1@mri.tum.de (P.M.); leonard.steinbacher@mri.tum.de (L.S.); samknoe@gmail.com (S.K.); haydar.kuekrek@mri.tum.de (H.K.); hans-guenther.machens@mri.tum.de (H.-G.M.); 2 Department of Dermatology, University Hospital Erlangen, Friedrich-Alexander University of Erlangen-Nürnberg, 91054 Erlangen, Germany; caihong.li@fau.de; 3 Department of Plastic, Reconstructive and Aesthetic Surgery, Isar Klinikum, 80331 Munich, Germany; ulf.dornseifer@isarklinikum.de

**Keywords:** hair loss, androgenetic alopecia, alopecia areata, dietary patterns, processed foods, antioxidant-rich foods

## Abstract

Background: Androgenetic alopecia (AGA) and alopecia areata (AA) impose significant psychosocial burdens. While pharmacological and surgical treatments exist, the role of dietary factors remains underexplored due to methodological limitations in observational studies. This Mendelian randomization (MR) study investigates causal relationships between 187 dietary exposures and hair loss, leveraging genetic variants to address confounding biases. Methods: Genome-wide association study (GWAS) data from 161,625 UK Biobank participants were analyzed, focusing on food preferences and intake patterns. Genetic instruments for each of the 187 dietary exposures were selected at a genome-wide significance threshold (*p* < 5 × 10^−8^), with rigorous sensitivity analyses (MR-Egger, MR-PRESSO) to validate causality. Outcomes included AA and AGA datasets from the FinnGen consortium. Results: MR analysis identified 18 specific dietary exposures significantly associated with non-scarring hair loss (FDR < 0.05). Protective effects emerged for antioxidant-rich dietary exposures, represented by higher preferences for melon, onions, and tea. Elevated risks were observed for certain exposures, including croissants, goat cheese, and whole milk. Alcohol consumption exhibited the strongest risk associations. Our extensive analysis of alcohol intake, combining data from multiple studies, consistently identified it as a significant risk factor for both alopecia areata and androgenetic alopecia. Conclusions: These findings imply modifiable dietary patterns in hair loss pathophysiology. A dual strategy is proposed: prioritizing polyphenol-rich plant foods while minimizing pro-inflammatory triggers like processed carbohydrates and alcohol. Clinically, tailored dietary adjustments—reducing ultra-processed foods and alcohol—may complement existing therapies for hair loss management.

## 1. Introduction

Hair loss disorders affect nearly half of the world’s adult population and rank among the most common dermatological consultations in primary care. Androgenetic alopecia (AGA) alone is estimated to affect up to 80% of men and 50% of women by 70 years of age, whereas alopecia areata (AA) accounts for approximately 2% of all outpatient appointments in dermatology clinics worldwide [1,2]. Alopecia exhibits significant bidirectional associations with psychological comorbidities: meta-analyses confirmed a 2.5-fold increased anxiety risk and a 2.7-fold depression risk versus controls [3], while a large cohort study identified persistent anxiety links across all age groups [4]. Systemic reviews emphasized that AA-induced psychosocial burden necessitates integrated clinical management, including psychological support as adjuvant care [5]. Recent epidemiological studies highlight that AGA not only impacts self-esteem but is also associated with comorbidities such as metabolic syndrome and cardiovascular diseases, suggesting systemic interactions between hair loss and overall health [6]. Current first-line therapies—including minoxidil, finasteride, and hair transplantation—face significant limitations. Pharmacological options are hindered by side effects (e.g., finasteride’s 1.57-fold increased risk of sexual dysfunction) and poor long-term adherence due to indefinite use requirements [7,8]. Surgical interventions, while effective, remain cost-prohibitive and fail to target underlying pathophysiology [9,10]. These limitations underscore the urgent need to identify modifiable risk factors, particularly dietary patterns, that could complement existing therapies.

Emerging epidemiological studies highlight dietary patterns as potential modifiable risk factors. For example, the Mediterranean diet—rich in antioxidants and unsaturated fats—has been inversely associated with AGA severity in Southern European cohorts [11]. Conversely, clinical evidence from anthropometric measurements and nutritional biomarkers demonstrated that Western diets characterized by ultra-processed foods correlate with earlier onset of non-scarring alopecia. [12]. Dietary factors modulate hair follicle biology through systemic pathways: chronic hyperglycemia from high-glycemic diets disrupts insulin signaling, exacerbating follicular miniaturization and upregulating androgen synthesis in predisposed individuals [13], while pro-inflammatory diets elevate oxidative stress and may trigger autoimmune responses [14]. However, observational studies face critical limitations: confounding by socioeconomic factors, reverse causality (e.g., stress-induced dietary changes in patients), and inaccuracies in self-reported dietary data. A systematic review indicates that socioeconomic factors, such as income, education, and occupation, significantly influence dietary patterns by shaping access to and affordability of nutrient-rich foods [15]. These challenges obscure causal relationships and hinder clinical translation [16].

Mendelian randomization (MR) offers a robust framework to circumvent these limitations by leveraging genetic variants as instrumental variables for dietary exposures [17]. Previous MR studies on diet and hair loss have largely focused on isolated nutrients or simplistic dietary indices, as exemplified by an antioxidant–alopecia investigation that tested only six micronutrients while neglecting the complexity of whole food consumption patterns, culinary practices, and nutrient interactions [18]. This narrow scope limits the applicability of findings to real-world dietary habits, which are inherently multidimensional [19]. From a genetic perspective, we hypothesize that specific dietary exposures have causal effects on non-scarring alopecia risk, as inferred through genetic variants serving as instrumental variables. To test this hypothesis, we conduct a large-scale Mendelian randomization study with three primary objectives: to comprehensively assess causal relationships between 187 dietary exposures and AGA/AA using genome-wide association data; to identify robust protective and risk-associated dietary factors; and to establish a foundation for tailored nutritional strategies that complement existing therapies.

## 2. Materials and Methods

### 2.1. Study Design

This study employed a two-sample Mendelian randomization (MR) approach as the primary method to investigate potential causal relationships between dietary exposures (primarily food preferences and patterns) and non-scarring hair loss phenotypes, including alopecia areata (AA) and androgenetic alopecia (AGA). MR leveraged genetic variants as instrumental variables to mitigate confounding and reverse causality inherent in observational data. To complement the MR analysis and validate findings for alcohol consumption—a key exposure with robust external data—we conducted a meta-analysis of effect estimates from multiple GWAS datasets on alcohol intake. This dual approach was adopted because comparable large-scale GWAS summary statistics were publicly available only for alcohol, allowing for external validation, whereas such data were inaccessible for the other exposures.

### 2.2. Data Sources

This study utilized data from the UK Biobank. It provides extensive phenotypic and genotypic data, including detailed dietary information derived from the UK Biobank food preferences web questionnaire [20]. Unlike conventional Food Frequency Questionnaires (FFQs) or 24 h recalls—which depend on memory and are vulnerable to recall and social desirability bias—the Food Preferences Questionnaire captures intrinsic liking, is largely independent of health-related self-presentation, and shows markedly higher test–retest reliability (≈0.8–0.9 vs. 0.5–0.8 for FFQs) [21]. Because adult food preferences remain stable over time, the resulting data enable robust temporal alignment with health outcomes. This rich dataset enabled us to assess a wide range of dietary exposures, from specific foods like fruits and vegetables to broader categories such as processed meats and beverages. Genome-wide association study (GWAS) statistics for dietary exposures were derived from May-Wilson’s study (*n* = 161,625), encompassing 139 food-liking traits quantified via a validated 9-point Likert scale. Structural equation modeling grouped these into 187 non-redundant dietary patterns, including “preference for grilled meats” and “frequency of fermented beverage consumption. These dietary patterns were derived via hierarchical structural equation modeling (SEM) based on genetic correlations between food-liking traits. Clustering was carried out using Ward’s D2 method, followed by confirmatory factor analysis with fit criteria (CFI > 0.9, SRMR < 0.1). Factors with genetic correlation > 0.9 were merged to ensure non-redundancy, resulting in a 4-level hierarchical map. The complete list of patterns, their constituent traits, and hierarchical relationships are publicly available at https://osf.io/e43x5/ (accessed on 1 July 2025) [22].

**Figure 1:**
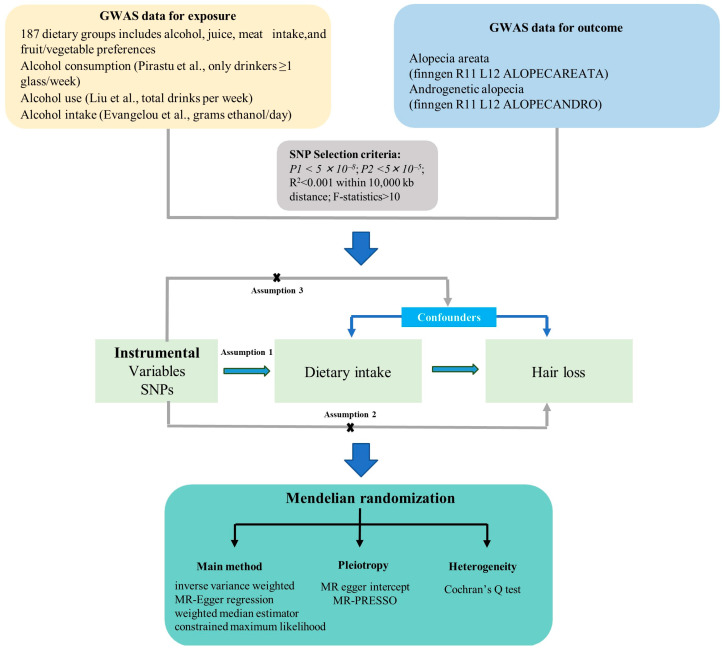
Flowchart of MR analysis methods in this research [23,24,25].

Additional actual alcohol consumption behaviors were sourced from complementary GWAS datasets: Pirastu et al. (*n* = 141,145) examined beverage-specific intake, including only participants who reported drinking ≥1 glass per week [23]; Liu et al. (*n* = 941,280) captured total intake with the composite “drinks per week” metric that incorporates both frequency and usual quantity [24]; and Evangelou et al. (*n* = 480,842) quantified exposure continuously as grams of ethanol consumed per day [25]. This integrated approach leveraging divergent yet complementary assessments of alcohol exposure provided a more comprehensive framework for understanding how drinking behavior affects disease risk. To minimize population stratification, single-nucleotide polymorphisms (SNPs) were restricted to those with a minor allele frequency (MAF) >1% in European ancestry individuals, and palindromic variants were excluded using 1000 Genomes Phase 3 reference data [26]. GWAS data on alopecia areata and androgenetic alopecia, derived from the R11 version of the finngen database, were used as outcomes [27]. Using these data, we conducted a Mendelian randomization (MR) analysis to explore potential causal relationships between dietary habits and hair loss-related phenotypes. The comprehensive research framework is illustrated in Figure 1.

### 2.3. Instrumental Variable (IV)

We established a *p*-value threshold of less than 5 × 10^−8^ to select relevant SNPs as instrumental variables for this study. This stringent threshold minimizes false positives, though it may exclude SNPs with modest but biologically relevant effects. To prevent linkage disequilibrium among the selected instrumental variables (IVs), we set the linkage disequilibrium correlation coefficient to R^2^ < 0.001 and the clumping distance to over 10,000 kb. The 10,000 kb window accounts for recombination hotspots, ensuring independence between selected SNPs. Additionally, palindromic and unproxied SNPs were removed from our analysis. The strength of the IVs was rigorously assessed using the F-statistic, a measure reflecting the proportion of variance in the exposure explained by the IVs and the sample size. SNPs with an F-statistic <10 were discarded to mitigate weak instrument bias, which could otherwise compromise the reliability of causal estimates [28].

### 2.4. MR Analysis

Mendelian randomization exploits the random allocation of genetic variants at conception to infer causality from observational data. In this research, the inverse variance weighted (IVW) test [29], MR-Egger regression [30], weighted median estimator [31], constrained maximum likelihood [32], and MR-PRESSO [33] were utilized for MR analysis. Among these methods, the IVW method can provide precise inference of causal relationships during the analysis process [34]. The IVW method is considered the primary basis for testing causal relationships in two-sample Mendelian randomization analyses. For traits characterized by a single SNP, we utilized the Wald ratio test to assess causal effects [35]. The remaining methods were used as auxiliary approaches to correct for pleiotropy. The results obtained from all methods were carefully considered to strengthen the evidence for causal relationships.

We employed MR-PRESSO and MR-Egger regression tests to investigate potential horizontal pleiotropic effects. A *p*-value < 0.05 for the intercept indicates the presence of horizontal pleiotropy. For the results of MR-PRESSO, we removed the SNPs identified as outliers and re-conducted the MR analysis. The MR Steiger directionality test was implemented to filter and remove SNP data with reverse causality [36]. To assess heterogeneity among the selected SNPs, we utilized Cochran’s Q statistic. These complementary analyses allowed for a comprehensive evaluation of the robustness and reliability of our causal inferences.

### 2.5. Meta-Analysis

We chose three publicly accessible datasets on alcohol consumption to conduct Mendelian randomization analysis on hair loss, employing the effect estimates derived from the IVW method as primary outcomes. For other dietary exposures, comparable external datasets were inaccessible; thus, meta-analytic validation was restricted to alcohol. The recommended metrics for effect estimates were 95% confidence intervals (CIs) and odds ratios (ORs). If the confidence interval range was wide, the results were presented using beta (β) and standard error (SE). Heterogeneity was evaluated using the Q test and *I*^2^ statistic. If heterogeneity reached statistical significance (*p* < 0.1 or *I*^2^ > 50%), a random-effects model was employed. In other cases, a fixed-effects model was applied [37]. Forest plots were generated to visualize effect sizes and confidence intervals across studies.

TwoSampleMR, MR-PRESSO, meta, and forestplot packages in R software (version 4.2.2) were used in the statistical analysis.

## 3. Results

### 3.1. SNP Selection

A large-scale GWAS on food preferences utilized a 9-point scale to assess liking for 139 specific foods and employed structural equation modeling combined with genetic correlations to identify 187 datasets. These datasets included detailed information such as alcohol and juice intake, various meats, and preferences for vegetables and fruits.

Using a significance threshold of *p* < 5 × 10^−8^, we identified 1551 SNPs as instrumental variables (IVs) based on our selection criteria. These SNPs were selected based on their strong association with dietary exposures and were confirmed to have F-statistics >10, ensuring robust instrument strength. For hair loss outcomes, SNPs were extracted from the FinnGen GWAS for AA and AGA (Supplementary Tables S1 and S2).

### 3.2. Dietary Liking and Alopecia Areata

**Figure 2:**
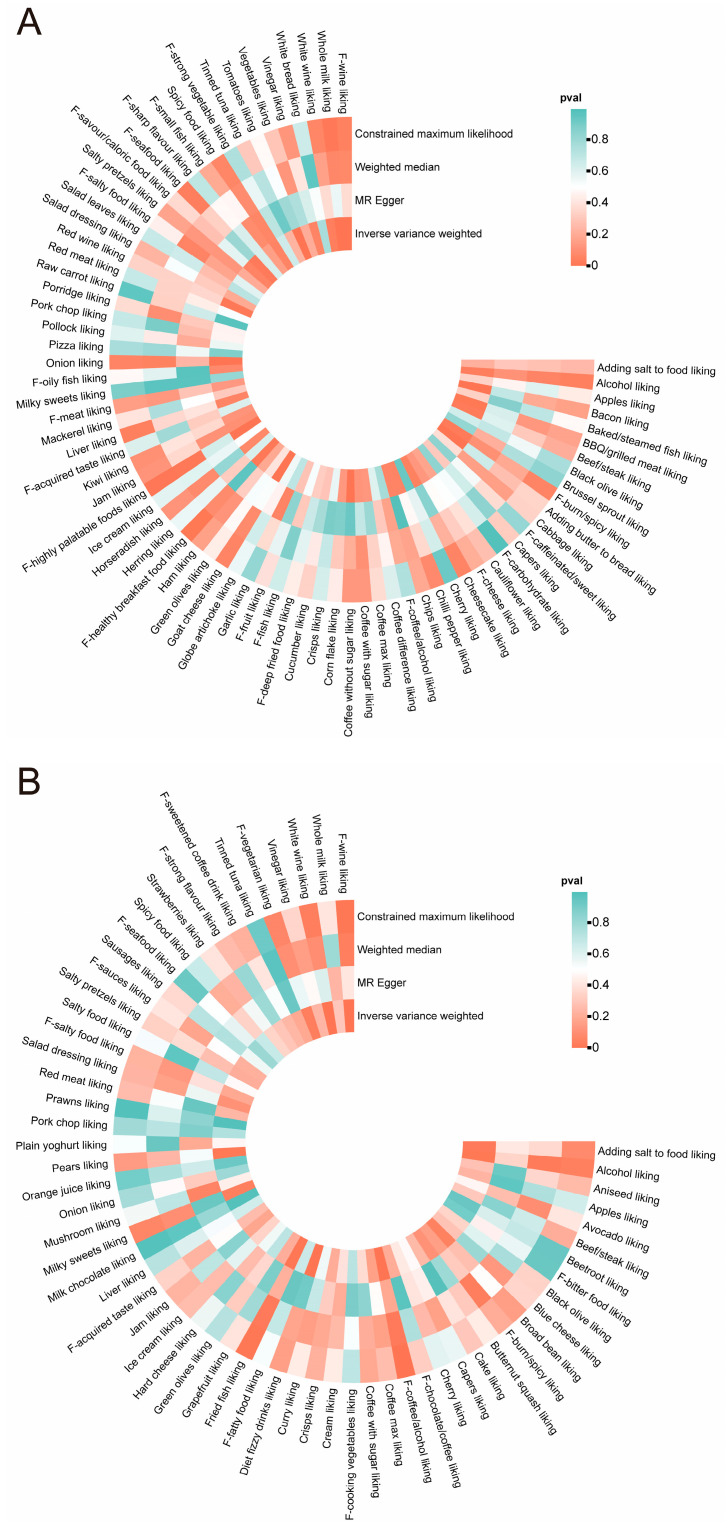
Circular heatmaps presenting the outcomes of four MR methods applied to 187 dietary preferences associated with hair loss. (**A**) Alopecia areata; (**B**) androgenetic alopecia.

**Figure 3:**
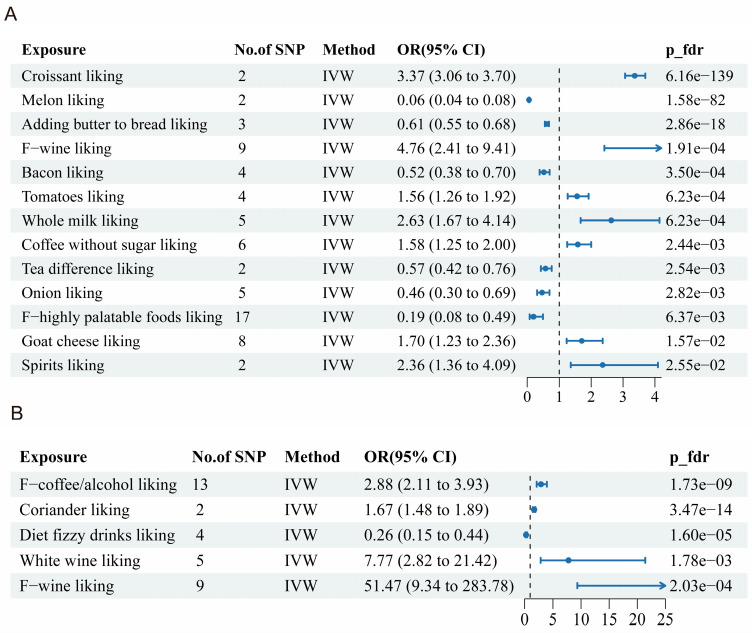
Forest plots showing the outcomes of the MR analysis using the IVW method. (**A**) Alopecia areata; (**B**) androgenetic alopecia.

MR analysis revealed significant associations between dietary preferences and alopecia areata (Figure 2A). After false discovery rate (FDR) correction, six protective factors and seven risk factors were identified (Figure 3A). Protective factors included melon (OR = 0.06, 95% CI: 0.04–0.08), buttered bread (OR = 0.61, 95% CI: 0.55–0.68), bacon (OR = 0.52, 95% CI: 0.38–0.70), tea (OR = 0.57, 95% CI: 0.42–0.76), onions (OR = 0.46, 95% CI: 0.30–0.69), and highly palatable foods (OR = 0.19, 95% CI: 0.08–0.49). The MR analysis showed a strong protective association between genetically proxied melon liking and alopecia areata. Only two independent lead variants—rs10275706 and rs80202633—served as instruments, jointly explaining just 0.0012 of the phenotypic variance. Although the corresponding F-statistics (97 and 90) exceed the conventional weak-instrument threshold, the exceptionally low R^2^ value (0.0012) suggests that while a causal protective effect is indicated, the extreme magnitude of the OR (0.06) should be interpreted with caution as very weak instruments can lead to an inflated or highly sensitive effect size estimate. This likely reflects scaling bias rather than a true biological effect magnitude. Risk factors included croissants (OR = 3.37, 95% CI: 3.06–3.70), wine (OR = 4.76, 95% CI: 2.41–9.41), tomatoes (OR = 1.56, 95% CI: 1.26–1.92), whole milk (>3.6 g fat per 100 g) (OR = 2.63, 95% CI: 1.67–4.14), unsweetened coffee (OR = 1.58, 95% CI: 1.25–2.00), goat cheese (OR = 1.70, 95% CI: 1.23–2.36), and spirits (OR = 2.36, 95% CI: 1.36–4.09). Notably, wine consumption exhibited the strongest risk association, highlighting its potential role in exacerbating alopecia areata.

### 3.3. Dietary Liking and Androgenetic Alopecia

Figure 2B illustrates the connections between dietary factors and androgenetic alopecia, with seven potential causal relationships outlined in Figure 3B. Risk factors included coffee/alcohol (OR = 2.88, 95% CI: 2.11–3.93), coriander (OR = 1.67, 95% CI: 1.48–1.89), white wine (OR = 7.77, 95% CI: 2.82–21.42), and F-wine (OR = 51.47, 95% CI: 9.34–283.78), while diet fizzy drinks showed a protective effect (OR = 0.26, 95% CI: 0.15–0.44). These findings underscore the detrimental impact of alcohol-related dietary habits on androgenetic alopecia.

The notably wide confidence intervals for F-wine (OR = 51.47) and other alcohol-related traits likely stem from inherent behavioral heterogeneity and the low proportion of variance explained by their genetic instruments (low R^2^). Importantly, the extreme effect size for F-wine (OR = 51.47) warrants cautious interpretation due to methodological considerations: this composite trait integrates white and red wine liking datasets, a process that may introduce modeling complexity. Combined with minimal variance explained (R^2^ = 0.003), such weak instruments disproportionately amplify odds ratios in MR frameworks; potential overfitting from hierarchical clustering could further inflate genetic signals. These findings underscore alcohol-related habits as risk factors for androgenetic alopecia, though precise effect magnitudes require validation through objective consumption metrics.

Therefore, to validate and sharpen our findings, we then performed a meta-analytic Mendelian randomization using GWAS data for actual alcohol consumption. This approach allowed us to test whether genetically predicted intake levels—rather than liking phenotypes—causally influence the risk of alopecia.

### 3.4. Sensitivity Analyses

Cochran’s Q test indicated no significant heterogeneity (*p* > 0.05) (Supplementary Tables S3 and S4). The MR-Egger intercept analysis showed no evidence of directional pleiotropy between dietary liking and alopecia (Supplementary Tables S5 and S6). Furthermore, the MR-PRESSO analysis (Supplementary Tables S7 and S8) detected no outliers associated with the outcomes. The precise *p*-values and intercept estimates are provided in the corresponding Supplementary Tables.

### 3.5. Meta-Analysis Results of Alopecia Areata

**Figure 4:**
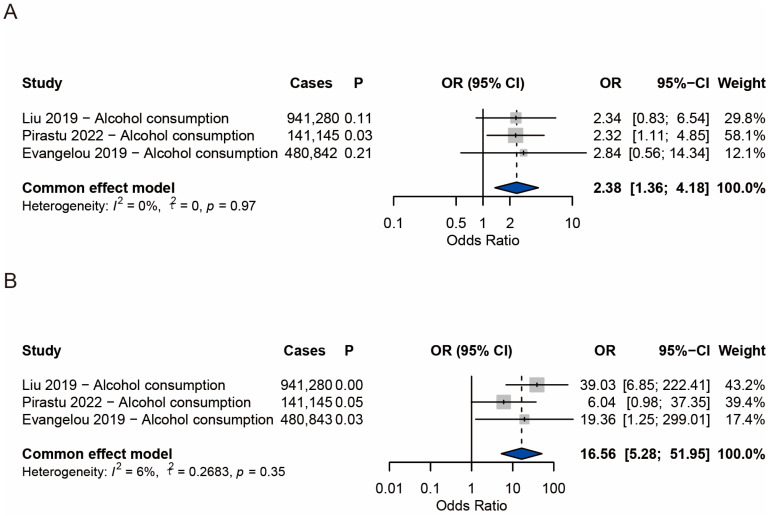
Forest plots illustrating the meta-analysis results regarding the association between alcohol consumption and alopecia [23,24,25]. (**A**) Alopecia areata; (**B**) androgenetic alopecia.

A meta-analysis of three studies examining alcohol consumption and alopecia areata yielded a pooled odds ratio of 2.38 (95% CI: 1.36–4.18; *p* = 0.0025; Figure 4A). Between-study heterogeneity was negligible (*I*^2^ = 0%, τ^2^ = 0; Q = 0.03, *p* = 0.97), justifying the use of a fixed-effects model.

### 3.6. Meta-Analysis Results of Androgenetic Alopecia

For androgenetic alopecia, three studies were included. The pooled odds ratio for the association with alcohol consumption was 16.56 (95% CI: 5.28–51.95; *p* < 0.0001; Figure 4B). Heterogeneity remained very low (*I*^2^ = 6%, τ^2^ = 0.2683; Q = 2.12, *p* = 0.35), supporting a fixed-effects model. Despite the large point estimate and wide confidence interval, the consistency across studies lends weight to a strong positive association.

## 4. Discussion

This study analyzed the intrinsic causal relationships between daily dietary intake and both alopecia areata and androgenetic alopecia from a genetic perspective. Based on Mendelian randomization, we identified 18 types of daily diets that either protect against or pose risks for hair loss. Our findings indicate that alcohol consumption and processed foods, such as croissants, are associated with an increased risk of hair loss, while antioxidant-rich foods, including melons, onions, and tea, confer protective effects. These results not only advance our understanding of hair loss etiology but also highlight actionable dietary modifications that could reduce disease burden.

Our most significant finding was that alcohol consumption significantly increased the risk of alopecia areata and androgenetic alopecia. Initially, we observed that within the 187 dietary groups, the OR values from the IVW results for the F-wine liking group were notably higher than those for other risk factors. To further confirm this finding, we included additional GWAS datasets on alcohol consumption in a meta-analysis, which enhanced the robustness of the conclusions. Some studies reported wide 95% confidence intervals for alcohol intake, likely attributable to the varying levels of alcohol consumption among the surveyed populations. Genetically, alcohol intake was consistently supported as a risk factor that accelerated hair loss. There was no evidence to suggest that moderate alcohol consumption could be a protective factor against hair loss [38]. Additionally, Mendelian randomization ruled out the impact of reverse causation, indicating that hair loss does not lead to a propensity for alcohol abuse. This finding provides a new perspective on the association between hair loss and alcohol use observed in clinical reports [39,40,41]. Alcohol consumption is associated with multiple mechanisms that may promote hair loss, including nutrient malabsorption (e.g., zinc and biotin), hormonal imbalances, and liver dysfunction [42]. Zinc deficiency, in particular, is a well-established cause of telogen effluvium, as zinc is critical for keratin synthesis and follicular proliferation, while alcohol-induced oxidative stress may exacerbate follicle damage by depleting antioxidant reserves. Additionally, alcohol’s dehydrating effects could compromise scalp hydration, further weakening hair structures [43]. The pronounced risk associated with wine and spirits in our study suggests that alcohol reduction could be a priority intervention for at-risk individuals.

Croissants exemplify high-temperature baked foods where the Maillard reaction between reducing sugars and free amino acids may generate acrylamide—a water-soluble compound with recognized toxicological profiles [44]. While dietary acrylamide exposure has been associated with carcinogenic and neurotoxic effects in experimental models [45], its potential role in alopecia pathogenesis is currently speculative and remains to be investigated. Notably, processed foods also tend to contain trans fats, refined sugars, and preservatives that can promote systemic inflammation [46], a process implicated in the autoimmune activity and follicular miniaturization characteristic of AA [47]. These considerations suggest that limiting processed food consumption might be a prudent component of holistic hair loss prevention, although the specific role of acrylamide in AA remains to be confirmed by targeted mechanistic studies.

Compared to skimmed milk and soy milk in 187 diet groups, cheese and whole milk contain higher levels of fat and cholesterol. Morinaga reported that stress induced by obesity, particularly from a high-fat diet, directly targeted hair follicle stem cells, leading to accelerated hair thinning [48]. Hao also found that increased dietary fat leads to hair loss phenotypes in mice [49]. Changes in cholesterol levels were often associated with hair disorders, including hair loss and hirsutism [50]. High-fat dairy products, such as whole milk and cheese, may exacerbate AGA by increasing circulating levels of dihydrotestosterone (DHT), a potent androgen that drives follicular miniaturization. However, direct human evidence specifically linking dietary fat intake to increased DHT levels and subsequent AGA exacerbation remains limited and warrants further investigation. Some studies suggested that the combined use of statins, such as simvastatin and ezetimibe, could reverse hair loss in patients with alopecia areata and prevent relapse [51,52]. Patients with alopecia areata are advised to avoid excessive fat and cholesterol in their daily diet.

Foods rich in antioxidants, such as melons and tea, may protect hair follicles by neutralizing reactive oxygen species (ROS), which are known to disrupt the hair growth cycle [53]. Polyphenols (e.g., epigallocatechin gallate in green tea) are known to stimulate hair growth [54]. Similarly, onions, rich in sulfur compounds and flavonoids (e.g., quercetin), also contribute to follicular protection [55,56]. These compounds have demonstrated antioxidant properties, increasing serum antioxidant capacity and reducing oxidative stress markers [57,58]. These protective mechanisms suggest that incorporating such foods into daily diets could bolster hair resilience against environmental and physiological stressors.

Our findings on the protective role of antioxidant-rich foods are consistent with previous studies demonstrating the benefits of vitamins C and E, as well as polyphenols, in reducing oxidative damage to hair follicles [10,59]. Similarly, frequent consumption of processed foods may exacerbate autoimmune pathogenesis through diet-induced systemic inflammation [60,61]. The strong risk association with alcohol consumption, particularly wine, is noteworthy, as observational studies have yielded mixed results [38,62]. Our MR approach supports a causal role for alcohol, overcoming limitations of prior research by minimizing confounding and establishing alcohol as a modifiable risk factor for both AGA and AA. This clarity strengthens the case for targeted dietary interventions in clinical practice.

This study benefits from several strengths, including the use of large-scale GWAS data, comprehensive assessment of 187 dietary exposures, and robust MR methods that account for pleiotropy and heterogeneity. However, limitations must be acknowledged. First, the reliance on European populations may limit the generalizability of our findings to other ethnic groups. Furthermore, significant cultural differences in dietary practices exist even within Europe and globally. These differences, along with distinct genetic backgrounds, mean that genetic predispositions, habitual food choices, and the cultural context influencing “food liking” may vary substantially. Second, the study population characteristics present limitations. The UK Biobank participants were aged 40–69 years at recruitment, limiting the applicability of our findings to younger or older adult populations where diet–health dynamics may differ. Third, food liking, as measured in the UK Biobank, may not fully capture actual dietary intake. The healthy volunteer bias inherent in the UK Biobank cohort (where participants are generally healthier and more health-conscious) may introduce systematic error into the reporting of food preferences, potentially leading to underestimation or overestimation of true dietary effects. Fourth, for the novel causal associations identified (e.g., croissants, bacon, and whole milk), the absence of publicly available large-scale GWAS summary statistics precluded external meta-analytic validation. Fifth, although MR minimizes confounding, residual concerns remain, including population stratification and confounding from psychological factors such as stress and anxiety. These conditions may influence both dietary choices and disease risk, potentially creating spurious associations captured by our genetic instruments and thus biasing the causal estimates.

Future research should prioritize several key directions to advance our findings. First, validation in diverse populations (e.g., Asian and African cohorts) is essential to assess the universality of dietary influences on alopecia and ensure equitable translation of dietary recommendations. Second, mechanistic studies must resolve the biological pathways linking diet to hair loss. Critically, the mediating role of hormonal factors requires targeted investigation. For androgenetic alopecia (AGA), where dihydrotestosterone (DHT) is a well-established pathogenic driver, multivariable Mendelian randomization leveraging genetic instruments for serum androgens should test whether dietary effects (e.g., high-fat dairy or alcohol) operate partly through the modulation of androgen metabolism or activity. For alopecia areata (AA), the interplay between diet, immune–endocrine axes, and autoimmune activation warrants exploration through integrated omics approaches. Third, translational work should bridge causal evidence to clinical practice. Dose–response analyses of protective nutrients (e.g., polyphenols in tea and sulfur compounds in onions) will define optimal intake thresholds. Randomized trials testing dietary interventions (e.g., alcohol reduction + antioxidant-rich food supplementation) must evaluate efficacy in halting hair loss progression.

Additionally, future MR studies should endeavor to address potential confounding from psychological factors such as stress and anxiety. This could involve leveraging genetic instruments for well-defined psychological traits in multivariable MR analyses to explicitly adjust for their effects. Alternatively, conducting MR studies within cohorts where psychological data is rigorously collected could facilitate sensitivity analyses to assess the robustness of dietary associations after accounting for psychological distress.

Ultimately, a systems-level framework integrating broader multi-omics layers—including genomics, metabolomics, proteomics, microbiome–host interactions, and, crucially, hormonal profiles—will be essential to fully decode the diet–hair axis. Mapping how dietary patterns remodel the diet–microbiome–endocrine–hair follicle ecosystem (e.g., via microbial metabolites or hormone–microbe crosstalk) will reveal novel therapeutic nodes. This convergence of evidence will accelerate precision–nutrition strategies tailored not only to host genetics and microbiota but also to endocrine and metabolic profiles, enabling truly personalized alopecia prevention and management.

## 5. Conclusions

This Mendelian randomization study provides supportive evidence for the causal role of dietary patterns in non-scarring hair loss, directly addressing our objectives to comprehensively assess causal relationships between 187 dietary exposures and alopecia areata (AA)/androgenetic alopecia (AGA). It revealed protective and risk-associated factors and established a foundation for tailored nutritional strategies that complement existing therapies. By leveraging genome-wide association data, we found 18 specific dietary exposures significantly associated with non-scarring hair loss, with protective effects from antioxidant-rich items such as melons, onions, and tea, alongside elevated risks from pro-inflammatory triggers including croissants, goat cheese, whole milk, and alcohol. Notably, alcohol emerged as the strongest risk factor across both AA and AGA, a signal corroborated by an integrated meta-analysis across independent alcohol consumption GWAS. These findings suggest that reducing the consumption of ultra-processed foods and alcohol, while increasing the intake of antioxidant-rich foods such as fruits, vegetables, and teas, may serve as a cost-effective adjunct to existing therapies for hair loss management. These dietary adjustments are accessible to most individuals, offering a low-risk strategy to complement pharmacological or surgical interventions. Given the high prevalence and psychosocial burden of hair loss disorders, these results advocate the integration of dietary guidelines into personalized dermatological care. By empowering patients with actionable lifestyle changes, clinicians can address both the physical and emotional aspects of hair loss, potentially improving quality of life and reducing healthcare costs. Further research in diverse populations and intervention trials is warranted to refine our understanding of the mechanisms involved and to develop evidence-based dietary recommendations for hair loss prevention and treatment, ensuring that these insights translate into tangible benefits for patients worldwide.

## Data Availability

This study was carried out using publicly available data from the finngen GWAS Project (https://www.finngen.fi/en, accessed on 1 July 2025) and Sebastian May-Wilson’s research (doi: 10.1038/s41467-022-30187-w).

## Supplementary Materials

The following supporting information can be downloaded at https://www.mdpi.com/article/10.3390/nu17152569/s1. The detailed data for this study are presented in the supplementary tables. Table S1: Instrumental variables used in MR analysis to assess the association between 187 diet groups and alopecia areata. Table S2: Instrumental variables used in MR analysis to assess the association between 187 diet groups and androgenetic alopecia. Table S3: The heterogeneity of Dietary liking instrumental variables with alopecia areata. Table S4: The heterogeneity of Dietary liking instrumental variables (androgenetic alopecia). Table S5: Directional horizontal pleiotropy assessed by intercept term in MR Egger regression (alopecia areata). Table S6: Directional horizontal pleiotropy assessed by intercept term in MR Egger regression (androgenetic alopecia). Table S7: MR-PRESSO analysis for the association between dietary liking and alopecia areata. Table S8: MR-PRESSO analysis for the association between dietary liking and androgenetic alopecia.
